# Pyrethroid Pesticide Exposure and Parental Report of Learning Disability and Attention Deficit/Hyperactivity Disorder in U.S. Children: NHANES 1999–2002

**DOI:** 10.1289/ehp.1308031

**Published:** 2014-09-05

**Authors:** Lesliam Quirós-Alcalá, Suril Mehta, Brenda Eskenazi

**Affiliations:** 1 Center for Environmental Research and Children’s Health, School of Public Health, University of California, Berkeley, Berkeley, California, USA; 2 Office of Children’s Health Protection, U.S. Environmental Protection Agency, Washington, DC, USA

## Abstract

Background: Use of pyrethroid insecticides has increased dramatically over the past decade; however, data on their potential health effects, particularly on children, are limited.

Objective: We examined the cross-sectional association between postnatal pyrethroid exposure and parental report of learning disability (LD) and attention deficit/hyperactivity disorder (ADHD) in children 6–15 years of age.

Methods: Using logistic regression, we estimated associations of urinary metabolites of pyrethroid insecticides with parent-reported LD, ADHD, and both LD and ADHD in 1,659–1,680 children participating in the National Health and Nutrition Examination Survey (1999–2002).

Results: The prevalence rates of parent-reported LD, ADHD, and both LD and ADHD were 12.7%, 10.0%, and 5.4%, respectively. Metabolite detection frequencies for 3-PBA [3-phenoxybenzoic acid], *cis*-DCCA [*cis*-(2,2-dichlorovinyl)-2,2-dimethylcyclopropane-1-carboxylic acid], and *trans*-DCCA [*trans*-(2,2-dichlorovinyl)-2,2-dimethylcyclopropane-1-carboxylic acid] were 77.1%, 35.6%, and 33.9%, respectively. The geometric mean 3-PBA concentration was 0.32 μg/L (median = 0.31 μg/L; interquartile rage = 0.10–0.89 μg/L). *cis*- and *trans*-DCCA 75th-percentile concentrations were 0.21 μg/L and 0.68 μg/L, respectively. Log_10_-transformed 3-PBA concentrations were associated with adjusted odds ratios (ORs) of 1.18 (95% CI: 0.92, 1.51) for parent-reported LD, 1.16 (95% CI: 0.85, 1.58) for ADHD, and 1.45 (95% CI: 0.92, 2.27) for both LD and ADHD. Adjusted ORs remained nonsignificant and decreased after controlling for creatinine and other environmental chemicals previously linked to altered neurodevelopment. Similarly, no significant associations were observed for *cis*- and *trans*-DCCA.

Conclusions: Postnatal pyrethroid exposure was not associated with parental report of LD and/or ADHD. Given the widespread and increasing use of pyrethroids, future research should evaluate exposures at current levels, particularly during critical windows of brain development.

Citation: Quirós-Alcalá L, Mehta S, Eskenazi B. 2014. Pyrethroid pesticide exposure and parental report of learning disability and attention deficit/hyperactivity disorder in U.S. Children: NHANES 1999–2002. Environ Health Perspect 122:1336–1342; http://dx.doi.org/10.1289/ehp.1308031

## Introduction

Pyrethroid insecticides account for > 30% of insecticides used worldwide (Barr et al. 2010). They are used to control pests in residential and agricultural settings, to treat head lice and scabies in humans and fleas in pets, for public health vector control, and for disinsection of commercial aircrafts [U.S. Environmental Protection Agency (EPA) 2013a, 2013b; Wei et al. 2012]. These synthetic insecticides act by altering the permeability of sodium ion channels in excited nerve cells, causing repetitive nerve impulses that may vary in intensity depending on the chemical structure of the individual pyrethroid (Barr et al. 2010; Mandhane and Chopde 1997; Nasuti et al. 2003). In humans, pyrethroid insecticides are rapidly metabolized and excreted (elimination half time: ~ 6–17 hr) [Agency for Toxic Substances and Disease Registry (ATDSR) 2003].

Use of pyrethroids, particularly in residential settings, has increased dramatically over the past decade and will likely increase further as they are replacing other pesticides (e.g., organophosphate pesticides) that are considered to have higher mammalian toxicity and are linked to adverse health effects in children (Barr et al. 2010; Horton et al. 2011a; U.S. EPA 2013b; Williams et al. 2008). Hence, exposure to pyrethroids in the U.S. general population is widespread (Barr et al. 2010) mostly from diet and indoor residential uses (via ingestion, dermal, and inhalation pathways) (ATDSR 2003; Lu et al. 2009). In the U.S. general population, higher pyrethroid exposures have been reported in children compared with adolescents and adults (Barr et al. 2010). Human exposure to pyrethroids is primarily assessed by measures of nonspecific urinary metabolites such as 3-phenoxybenzoic acid (3-PBA), and *cis-* and *trans*-(2,2-dichlorovinyl)-2,2-dimethylcyclopropane-1-carboxylic acid (*cis*- and *trans*-DCCA) (Barr et al. 2010).

Although pyrethroids are considered to be safer than other insecticides, rodent studies suggest that early-life and pubertal pyrethroid exposures alter neurobehavioral functioning (Farag et al. 2007; Shafer et al. 2005; Sinha et al. 2006). For example, one study reported that early prenatal and postnatal pyrethroid exposure led to oxidative stress in various areas of the brain, with the hippocampal area exhibiting cholinergic dysfunction. Neurochemical changes were accompanied by impaired learning and memory (Sinha et al. 2006). Another rodent study reported associations of pyrethroid exposure during puberty with spatial learning and memory impairments that were more severe in females, increased anxiety in females, and inhibition of aggressive behavior in males (Meng et al. 2011). Pubertal pyrethroid exposure has also been reported to disrupt testosterone and estradiol synthesis and expression of androgen receptor in the cerebral cortex, which may impair neurobehavioral development (Liu et al. 2011). Select pyrethroids have also shown greater toxicity in neonatal than in adult rats, possibly due to incomplete development of detoxifying enzymes (Cantalamessa 1993).

In humans, symptoms of systemic pyrethroid poisoning resulting from accidental exposure or intentional ingestion are well characterized (ATDSR 2003; Ray and Forshaw 2000; Soderlund et al. 2002). However, data on the human health effects of pyrethroids (particularly neurodevelopmental effects) at the lower environmental doses encountered by the general public are limited, and data on developing children are particularly sparse. Only two studies to date have evaluated the effects of prenatal pyrethroid exposure on children’s neurodevelopment. Horton et al. (2011b) examined the relationship of prenatal exposure to permethrin, a pyrethroid, and piperonyl butoxide (PBO), a synergist commonly formulated with pyrethroids, on children’s neurodevelopment at 3 years of age (*n* = 230–342). Children in the highest PBO exposure group, as assessed in personal air samples, scored approximately four points lower on the Bayley Mental Developmental Index than children in the lowest exposure group. Prenatal exposure to permethrin in air and/or blood was not associated with altered neurodevelopment, although the authors noted difficulty measuring permethrin in these media (Horton et al. 2011b). Another study reported an inverse association between prenatal pyrethroid exposure, using urinary metabolites, and measures of motor function, social adaptation, and intelligence in 1-year-old Chinese infants (*n* = 497) (Xue et al. 2013). Neither of these studies controlled for postnatal pyrethroid exposures.

Two other studies have considered the potential effects of childhood pyrethroid exposure on neurodevelopment. A study of 7- to 9-year-old Nicaraguan children (*n* = 110) living in an agricultural community (Rodríguez 2012) found that parent-reported hours of pyrethroid use during the first year of life was associated with poorer perceptual reasoning using the Wechsler Intelligence Scale for Children IV and teacher-reported hyperactivity and attention deficit/hyperactivity disorder (ADHD) using the revised Conner’s Teachers Rating Scale short version. In a subset of these children (*n* = 74), child’s 3-PBA urinary concentration levels were associated with impaired cognition and ADHD in girls but not boys (Rodríguez 2012). Most recently, a nationally representative Canadian study found an association between urinary concentrations of *cis*-DCCA and parent-reported total behavioral problems on the Strengths and Difficulties Questionnaire in 6- to 11-year-olds (*n* = 779). However, urine 3-PBA concentrations were not associated with behavioral problems in the study population (Oulhote and Bouchard 2013).

Herein, we examine the cross-sectional association between children’s pyrethroid exposures and parent-reported learning disability (LD) and/or ADHD in a representative sample of U.S. 6- to 15-year-olds participating in the National Health and Nutrition Examination Survey (NHANES). LDs encompass various difficulties in receptive and expressive language, reading, and mathematics, which may affect scholastic performance (Pastor and Reuben 2002); ADHD is a neurobehavioral disorder characterized by a persistent pattern of inattention, impulsivity, and/or hyperactivity that interferes with functioning or development (American Psychiatric Association 2013).

## Methods

*Data source and study population*. Data for this analysis were extracted from NHANES, a population-based, cross-sectional survey assessing the health and nutritional status of the U.S. civilian, noninstitutionalized population that is conducted by the National Center for Health Statistics (NCHS) of the U.S. Centers for Disease Control and Prevention (CDC). The sample is selected to represent the U.S. population of all ages, using a complex, stratified multistage probability sample design, which oversamples certain subpopulations. Information on study participants was collected via a household interview. A standardized physical examination, consisting of select medical and laboratory tests, was conducted in a mobile examination center. Study activities were approved by the NCHS institutional review board; parents/guardians provided written consent for their minor children (i.e., < 17 years) to participate, and children assented in the presence of the parent/guardian and interviewer (Zipf et al. 2013). Further details on interviews, examination procedures, and sample collection are available elsewhere (CDC 2014). For this analysis, we used publicly available data for children between 6 and 15 years of age who participated in two consecutive NHANES cycles (1999–2000 and 2001–2002). Pyrethroid exposure measurements were conducted on a random subsample of participants ≥ 6 years of age. We selected study cycles and child age range based on availability of exposure, outcome, and critical covariate data.

*Parental report of an LD and ADHD*. Our primary outcome variables, parental report of an LD and/or ADHD, were based on parental/guardian response to two NHANES interview questions: “Has a representative from a school or a health professional ever told (you) that (the child) had a learning disability?” and “Has a doctor or health professional ever told (you) that (the child) had attention deficit disorder?” For children who were ≥ 12 years of age, the same questions about LD and ADHD were asked; however, the words in parentheses were replaced with [the child] and [he/she], respectively. Question phrasing did not differ by cycle year. For this analysis, we classified children as reported with LD, ADHD, or with both LD and ADHD.

*Pyrethroid exposure assessment*. To assess pyrethroid exposure, we used urinary metabolite measurements of 3-PBA, *cis*-DCCA, and *trans*-DCCA in spot urine samples provided by participants during the physical examination. The metabolite 3-PBA represents exposure to permethrin, cypermethrin, deltamethrin, allethrin, resmethrin, fenvalerate, cyhalothrin, fenpropathrin, and tralomethrin; and *cis*- and *trans-*DCCA represent exposure to the *cis-* and *trans-* isomers, respectively, of permethrin, cypermethrin, and cyfluthrin (Barr et al. 2010; CDC 2009). Two other pyrethroid metabolites [4-fluoro-3-phenoxybenzoic acid (4F3PBA), a specific metabolite of cyfluthrin; and *cis*-(2,2-dibromovinyl)-2,2-dimethylcyclopropane-1-carboxylic acid (*cis-*DBCA), a specific metabolite of deltamethrin] were also measured; however, they were not included in our analyses due to low detection frequencies (≤ 3.2%). Samples were analyzed at the CDC’s National Center for Environmental Health laboratory and metabolite concentrations were measured through high-performance liquid chromatography/tandem mass spectrometry using validated laboratory methods detailed elsewhere (Baker et al. 2004; Olsson et al. 2004). The limit of detection (LOD) for 3-PBA and *cis-*DCCA was 0.10 μg/L, and 0.40 μg/L for *trans-*DCCA. Urinary creatinine (milligrams per deciliter) concentrations were determined using an automated colorimetric method based on a modified Jaffe reaction on a Beckman Synchron AS/ASTRA clinical analyzer (Beckman Instruments, Inc., Brea, CA) at the Fairview University Medical Center (Minneapolis, MN) (Barr et al. 2010).

Of the 4,672 children 6–15 years old surveyed during 1999–2002, only 1,861 (39.8%), 1,839 (39.4%), and 1,848 (39.6%) children had urinary 3-PBA, *cis-* and *trans*-DCCA data, respectively; two children with *cis-*DCCA, one child with *trans-*DCCA, and one child with 3-PBA data were missing creatinine data. Compared with children who did not have pyrethroid metabolite data, children who did were more likely to be of other races/ethnicities (i.e., multiracial or of other races/ethnicities besides non-Hispanic white, non-Hispanic black, or Mexican American), to have the head of household with an education level greater than high school, to not have attended a daycare or preschool, and to have no health insurance (see Supplemental Material, Table S1). Of the children with pyrethroid metabolite measurements, two were missing information on LD and five on ADHD.

Prevalence rates of LD, ADHD, and both LD and ADHD were similar between children with and without pyrethroid metabolite data (see Supplemental Material, Table S2). Additionally, outcome prevalence rates did not differ significantly (*p* ≥ 0.36) by study cycle (not shown). A total of 1,680 (90.3%), 1,659 (90.2%), and 1,669 (90.3%) children had complete exposure (3-PBA and *cis-* and *trans-*DCCA, respectively), outcome, and covariate data available for multivariable analysis.

*Statistical analyses*. We computed descriptive statistics [e.g., geometric means (GMs), GM 95th percent confidence intervals (CIs), and weighted percentiles] for 3-PBA concentrations and, given their low detection frequencies, weighted 75th percentiles for *cis-* and *trans-*DCCA. To assess associations between our main exposures, urinary pyrethroid metabolites, and each outcome of interest, we used logistic regression models to estimate crude and adjusted odds ratios (ORs) and corresponding 95% CIs. We constructed separate models for each metabolite to assess its association with parent-reported LD, ADHD, and both LD and ADHD. Concentrations of 3-PBA were log-normally distributed; thus, we modeled 3-PBA concentrations as a continuous log_10_-transformed variable in our analyses. Concentrations of 3-PBA below the analytical LOD (23% of values) were imputed at random in R (version 3.1.0; http://www.r-project.org/foundation/) based on a log-normal probability distribution whose parameters were determined by maximum likelihood estimation with the imputed values forced to be below the LOD. This method yields reasonable estimates when detection frequencies are > 70% (Lubin et al. 2004). Concentrations of *cis-* and *trans-*DCCA were dichotomized (detected vs. not detected) given their low detection frequency (35.6% and 33.9%, respectively).

For the multivariable models, children with parental report of the outcome of interest were compared with all others without the specific outcome modeled (e.g., in models of LD, the comparison population would include those with ADHD). We also ran models in which the comparison population did not include either LD or ADHD cases. Results were similar; thus, only the former models are shown.

We examined several variables as potential confounders for inclusion in our models. Variables retained *a priori* in all multivariable models included child’s sex, race/ethnicity (non-Hispanic white, non-Hispanic black, Mexican American, and other), child’s age (years), household reference person’s education level [did not graduate high school, graduated high school or received GED (General Educational Development) certification, and greater than high school education], and health insurance status (yes/no). Covariates that predicted at least one outcome of interest with *p* < 0.20 in separate bivariate analyses were included in all final models. Covariates considered included: birth weight (≥ 2,500 g vs. < 2,500 g), neonatal intensive care unit admission (NICU; yes/no); preschool or daycare attendance (yes/no); mother’s age at child’s birth (years); maternal smoking status during pregnancy (yes/no); and poverty income ratio (continuous). Because poverty income ratio (PIR) data were missing on 166 children, we retained health insurance status and household reference person’s education level (both associated with PIR at *p* < 0.0001) as proxies for socioeconomic status in all final models to maintain sample size. To ensure the validity of our findings, we ran separate analyses on the subsample with PIR as a covariate in the model; results did not change appreciably (see Supplemental Material, Table S3).

To account for the effects of urine dilution on metabolite concentrations, we ran separate models adjusted for creatinine concentration (milligrams per deciliter) as a covariate (Barr et al. 2010). We did not model creatinine-corrected metabolite concentrations because creatinine concentrations varied widely in our sample population (Barr et al. 2005). In separate analyses, we also examined potential sex-related differences in the association between urinary 3-PBA concentrations and our outcomes by introducing an interaction term for sex and urinary 3-PBA concentrations to our final models. We also assessed possible interaction between children’s age and 3-PBA concentrations, both as continuous variables.

In sensitivity analyses, we also considered in the models exposures to other agents purported to be neurotoxicants, including organophosphate pesticides as measured via nonspecific urinary dialkylphosphate metabolites [DAPs; comprised of the molar sum (nanomoles per liter) of dimethyl phosphate, diethyl phosphate, dimethylthiophosphate, diethylthiophosphate, dimethyldithiophosphate, and diethyldithiophosphate] (Bouchard et al. 2010, 2011), environmental tobacco smoke as measured using serum cotinine concentrations (nanograms per milliliter) (Froehlich et al. 2009), and lead as measured in children’s blood (micrograms per deciliter) (Lanphear et al. 2000). Because concentrations for these environmental contaminants were log-normally distributed in our population, we log_10_-transformed these concentrations. Concentrations of these chemicals below their LODs were assigned a concentration value of LOD divided by the square root of 2 (CDC 2009). We controlled for each environmental contaminant in separate models and then altogether in the same model. Inclusion of environmental contaminants decreased our final models’ sample size because of missing participant data. To determine whether missing participants were affecting estimates, we ran models with and without adjustment for each chemical among only those participants with data on each respective environmental chemical.

Although the main criteria for ADHD were based on parental report alone, we also considered in additional analyses children who were reported to use stimulant medication, to maximize the likelihood that ADHD cases were diagnosed by a medical professional. Of the 148 children with a parental report of ADHD, 61 children (41.2%) also reported using stimulant medication. Thus, we defined children with ADHD based on *a*) parental report of ADHD (*n* = 148 children met this criterion) (our main analyses), and in separate analyses based on *b*) parental report of ADHD or using stimulant medication (*n* = 154 children met this criterion), and *c*) parental report of ADHD and using stimulant medication (*n* = 61 children met this criterion). We did not look at children with no parental report but who were using stimulant medication because the sample size was too small (*n* = 6). Information on stimulant medication use was provided by respondents and was based on National Drug Codes corresponding to amphetamine aspartate/amphetamine sulfate/dextroamphetamine saccharate/dextroamphetamine sulfate (3700), dextroamphetamine sulfate (17900), methylphenidate hydrochloride (39500), or unspecified ADHD drug medications (82000).

Last, we also explored whether cohort effects were present by including a covariate for NHANES survey cycle year in our final adjusted models as part of our sensitivity analyses. Except for evaluating cohort effects, sensitivity analyses were limited to 3-PBA, given the low detection frequency of *cis-* and *trans-*DCCA.

NCHS-created sampling weights, strata, and primary sampling units were applied in all statistical analyses according to the NCHS guidelines to yield robust SEs and unbiased point estimates, and to account for the complex, stratified multistage probability sample design of NHANES. All data analysis was performed using SAS, version 9.3 (SAS Institute Inc., Cary, NC). The threshold for statistical significance in our logistic regression analyses (i.e., *p*-value on the main exposure variable) was set at *p* < 0.05 and at *p* < 0.10 for interactions.

## Results

The metabolite 3-PBA was detected in 77.1% of the urine samples, whereas lower detection frequencies were observed for *cis-* and *trans*-DCCA (35.6% and 33.9%, respectively). The GM urinary 3-PBA concentration was 0.32 μg/L (median = 0.31 μg/L; interquratile range = 0.10–0.89 μg/L). The weighted 75th percentile concentrations for *cis-* and *trans-*DCCA were 0.21 μg/L and 0.68 μg/L, respectively. Among children with detectable pyrethroid metabolite concentrations, we found that *cis-* and *trans-*DCCA concentrations were significantly correlated (*p* < 0.0001) with each other (*r* = 0.89) and with 3-PBA (*r* = 0.96 and *r* = 0.88 for *cis-* and *trans-*DCCA, respectively), suggesting that 3-PBA was more likely to be a marker of exposure to cypermethrin and/or permethrin (precursor pyrethroid pesticides common to these three urinary metabolites) rather than other pyrethroid insecticides.

Urinary concentrations of 3-PBA by participant characteristics are presented in Table 1. GM urinary 3-PBA concentrations were higher in children who were non-Hispanic black than in children of other races/ethnicities (GM = 0.43 μg/L vs. GM ≤ 0.34 μg/L). Additionally, children in the lowest PIR quartile had higher 3-PBA concentrations than children in the other three PIR quartiles (GM = 0.45 μg/L vs. GM ≤ 0.37 μg/L). Children living in homes with a head of household with less than a high school education also had higher 3-PBA concentrations than children in homes with a head of household with more education (GM = 0.43 μg/L vs. GM ≤ 0.33 μg/L). Significant but weak correlations were observed between concentrations of 3-PBA and each of the environmental chemicals considered (3-PBA and DAPs *r* = 0.12; 3-PBA and blood lead *r* = 0.12; and 3-PBA and serum cotinine *r* = 0.18, all *p* < 0.001).

**Table 1:** Urinary 3-PBA concentrations (μg/L) based on children’s characteristics (NHANES 1999–2002; *n* = 1,861).

Characteristic	*n* (%)	3-PBA concentration (μg/L) [GM (95% CI)]	*p*-Value^*a*^
Sex
Male	899 (51.7)	0.34 (0.28, 0.42)	0.24
Female	962 (48.3)	0.39 (0.32, 0.48)
Missing	0
Race/ethnicity
Non-Hispanic white	466 (58.5)	0.30 (0.23, 0.40)	0.001
Non-Hispanic black	596 (14.6)	0.43 (0.36, 0.53)
Mexican American	625 (11.3)	0.25 (0.20, 0.31)
Other	174 (15.7)	0.34 (0.27, 0.42)
Missing	0
PIR
< 0.94	549 (22.9)	0.45 (0.36, 0.56)	0.001
0.94–1.80	441 (24.0)	0.37 (0.27, 0.50)
1.81–3.56	387 (25.9)	0.33 (0.25, 0.45)
> 3.56	318 (27.2)	0.19 (0.15, 0.26)
Household reference person’s education level
< High school	688 (24.7)	0.43 (0.35, 0.53)	0.002
High school	438 (25.9)	0.33 (0.24, 0.44)
> High school	663 (49.4)	0.27 (0.21, 0.35)
Missing	72
Low birth weight (< 2,500 g)
Yes	160 (7.4)	0.31 (0.19, 0.51)	0.96
No	1,629 (92.6)	0.32 (0.26, 0.39)
Missing	72
Age (years)
6–7	333 (19.8)	0.34 (0.22, 0.52)	0.12
8–9	377 (20.4)	0.32 (0.25, 0.42)
10–11	339 (18.3)	0.27 (0.20, 0.35)
12–13	411 (20.6)	0.37 (0.27, 0.49)
14–15	401 (20.9)	0.29 (0.22, 0.40)
NICU
Yes	205 (12.0)	0.37 (0.25, 0.53)	0.38
No	1,641 (88.0)	0.31 (0.25, 0.38)
Missing	15
Attended day care/preschool
Yes	1,229 (71.6)	0.30 (0.24, 0.38)	0.22
No	630 (28.4)	0.36 (0.27, 0.47)
Missing	2
Health insurance
Yes	1,492 (82.8)	0.31 (0.25, 0.37)	0.32
No	348 (17.2)	0.37 (0.24, 0.56)
Missing	21
Total DAPs (mol/L)
< 3.15 × 10^–8^	517 (30.5)	0.25 (0.19, 0.32)	0.04
3.15 × 10^–8^ to 1.14 × 10^–7^	594 (31.1)	0.34 (0.27, 0.43)
> 1.14 × 10^–7^	718 (38.4)	0.36 (0.27, 0.47)
Missing	32
Blood lead level (μg/dL)
0.2–1.2	771 (50.1)	0.27 (0.22, 0.34)	0.003
1.3–2.1	522 (31.4)	0.41 (0.31, 0.54)
> 2.1	369 (18.5)	0.45 (0.32, 0.63)
Missing	199
Serum cotinine (ng/mL)
Bottom 50% (< 0.06)	650 (41.2)	0.21 (0.17, 0.28)	< 0.0001
Upper 50% (0.06–9.99)	910 (55.7)	0.47 (0.39, 0.55)
≥ 10	35 (3.1)	0.43 (0.23, 0.80)
Creatinine (mg/dL)
< 92	685 (38.8)	0.17 (0.14, 0.22)	< 0.0001
92–164	717 (39.3)	0.40 (0.30, 0.53)
> 164	459 (21.8)	0.56 (0.43, 0.73)
Missing	0
Mother’s age at child’s birth (years)
< 25	861 (41.8)	0.37 (0.35, 0.46)	0.1
25–34	836 (49.2)	0.29 (0.22, 0.37)
≥ 35	135 (9.0)	0.22 (0.13, 0.38)
Missing	29	—
Mother smoked during pregnancy
Yes	265 (18.7)	0.37 (0.28, 0.49)	0.12
No	1,566 (81.3)	0.31 (0.25, 0.38)
Missing	30
Sample includes children with 3-PBA and creatinine data. Percentages, GMs, and 95% CIs are weighted to reflect sampling. ^***a***^*t*-Test *p*-value comparing GMs among subgroups.			

Of the 1,861 children with 3-PBA and creatinine concentrations, 223 (12.7%), 148 (10.0%), and 78 (5.4%) had a parental report of LD, ADHD, and both LD and ADHD, respectively; similar results were observed for children with *cis-* and *trans-*DCCA data (see Supplemental Material, Table S2). Participants with a parental report of LD, ADHD, and both LD and ADHD had higher urinary 3-PBA concentrations than children without these conditions (Table 2). Specifically, non-creatinine-standardized GM 3-PBA concentrations were 0.42 μg/L (95% CI: 0.31, 0.57) in children reported having LD versus 0.30 μg/L (95% CI: 0.25, 0.37) for children without LD (*p* = 0.007); 0.35 μg/L (95% CI: 0.25, 0.49) in children with ADHD versus 0.31 μg/L (95% CI: 0.26, 0.38) for children without ADHD (*p* = 0.38); and 0.52 μg/L (95% CI: 0.32, 0.83) for children with parent-reported LD and ADHD versus 0.31 μg/L (95% CI: 0.25, 0.38) for children without both of these conditions (*p* = 0.02).

**Table 2:** Geometric mean 3-PBA concentrations (μg/L) based on outcomes for study participants (*n* = 1,861).

Neurodevelopmental outcome	*n* (%)	3-PBA concentration (μg/L) [GM (95% CI)]	*p*-Value^*a*^
LD
Yes	223 (12.7)	0.42 (0.31, 0.57)	0.007
No	1,636 (87.3)	0.30 (0.25, 0.37)
ADHD
Yes	148 (10.0)	0.35 (0.25, 0.49)	0.38
No	1,708 (90.0)	0.31 (0.26, 0.38)
LD + ADHD
Yes	78 (5.4)	0.52 (0.32, 0.83)	0.02
No	1,776 (94.6)	0.31 (0.25, 0.38)
Sample includes children with 3-PBA and creatinine data. Percentages, GMs, and 95% CIs are weighted to reflect survey sampling. Study participants with missing data: for LD (*n *= 2), ADHD (*n *= 5), and LD + ADHD (*n *= 7). ^***a***^*p*-Value comparing GMs between groups.			

Results from crude and multivariable logistic regression analyses for 3-PBA are presented in Table 3. For every 10-fold increase in 3-PBA concentrations, there were 1.24 (95% CI: 1.00, 1.55), 1.26 (95% CI: 0.96, 1.65), and 1.55 (95% CI: 1.04, 2.32) unadjusted ORs for children having a parental report of LD, ADHD, and both LD and ADHD, respectively, compared with children without parental report of these disorders. After taking into account potential confounders, the ORs became slightly attenuated: for every 10-fold increase in 3-PBA concentration, there were 1.18 (95% CI: 0.92, 1.51), 1.16 (95% CI: 0.85, 1.58), and 1.45 (95% CI: 0.92, 2.27) adjusted odds ratios (aORs) for children having a parental report of LD, ADHD, and both LD and ADHD, respectively, compared with children without parental report of these disorders. Results were further attenuated when creatinine concentrations were included in the models (Table 3). No interactions between 3-PBA exposure and sex or child age (i.e., all interaction *p*-values > 0.10) were observed (data not shown).

**Table 3:** Association between pyrethroid exposure and parent-reported LD, ADHD, and both LD and ADHD.

Neurodevelopmental outcome	cOR (95% CI)	*p*	aOR^*a*^ (95% CI)	*p*	aOR_creat_^*a*^ (95% CI)	*p*
3-PBA (*n *= 1,680)
LD	1.24 (1.00, 1.55)	0.05	1.18 (0.92, 1.51)	0.19	1.11 (0.83, 1.48)	0.49
ADHD	1.26 (0.96, 1.65)	0.10	1.16 (0.85, 1.58)	0.35	1.06 (0.76, 1.48)	0.72
LD + ADHD	1.55 (1.04, 2.32)	0.03	1.45 (0.92, 2.27)	0.11	1.31 (0.82, 2.09)	0.26
*cis-*DCCA (*n *= 1,659)
LD	1.58 (0.97, 2.58)	0.07	1.43 (0.83, 2.48)	0.20	1.36 (0.76, 2.43)	0.31
ADHD	1.08 (0.71, 1.64)	0.72	1.00 (0.64, 1.57)	0.99	0.90 (0.56, 1.45)	0.68
LD + ADHD	1.30 (0.59, 2.84)	0.51	1.23 (0.56, 2.70)	0.61	1.08 (0.47, 2.46)	0.86
*trans-*DCCA (*n *= 1,669)
LD	1.54 (0.92, 2.60)	0.10	1.46 (0.85, 2.50)	0.17	1.36 (0.74, 2.49)	0.33
ADHD	1.46 (0.86, 2.48)	0.16	1.29 (0.72, 2.33)	0.40	1.15 (0.62, 2.13)	0.65
LD + ADHD	2.04 (0.97, 4.29)	0.06	1.84 (0.88, 3.84)	0.11	1.62 (0.74, 3.56)	0.23
Abbreviations: aOR, adjusted odds ratio; aOR_creat_, adjusted odds ratio including creatinine in the model; cOR, crude odds ratio. Model sample sizes were based on the number of children with complete information on exposure, outcome, and covariates. Thus, estimates provided (i.e., cOR, aOR, and aOR_creat_) are based on the same set of observations indicated for each metabolite. Pyrethroid exposure was modeled as follows: for 3-PBA we used log_10_-transformed concentrations; for *cis*- and *trans-*DCCA we used a dichotomous variable (detected vs. not detected) given their low detection frequencies. Number of children with LD, ADHD, and LD + ADHD and a detectable concentration of *cis*-DCCA were 88, 49, and 26, respectively. Number of children with LD, ADHD, and LD + ADHD and a detectable concentration of trans-DCCA were 81, 54, and 32, respectively. ^***a***^Models were adjusted for sex, age, race/ethnicity, household reference education level, low birth weight status, maternal age at child’s birth, NICU admission, maternal smoking during pregnancy, day care/preschool attendance, and health insurance.						

When we controlled for other environmental chemicals (i.e., total DAPs, serum cotinine, and blood lead concentrations) in our sensitivity analyses, results remained similar albeit slightly attenuated (see Supplemental Material, Table S3).

Additionally, results remained nonsignificant when we extended our definition of ADHD to include those children with parent-reported ADHD or taking stimulant medication (aOR = 1.16; 95% CI: 0.85, 1.58; aOR including creatinine as a covariate, aOR_creatinine_ = 1.07; 95% CI: 0.77, 1.50), and to include children with parent-reported ADHD and taking stimulant medication (aOR= 0.79; 95% CI: 0.42, 1.51; aOR_creatinine_ = 0.72; 95% CI: 0.36, 1.42).

No significant associations were observed between any of our outcomes and detection of *cis-* and *trans-*DCCA (Table 3). As with 3-PBA, adjusted associations became further attenuated when creatinine was taken into account.

Last, although we did not observe significant differences in GM 3-PBA concentrations between the two NHANES cycles (1999–2001 vs. 2001–2002), we did observe significantly higher detection rates (*p* < 0.01) of *cis*- and *trans*-DCCA during the 1999–2001 cycle compared with the subsequent cycle (see Supplemental Material, Table S4). When we included survey cycle year in our final models, aORs generally increased slightly; however, results remained similarly nonsignificant for all three metabolites, and the survey cycle year covariate was not significant in any of the models (not shown).

## Discussion

Neurodevelopmental disorders such as LD and ADHD affect between 400,000 and 600,000 children in the United States each year. The disabilities caused by such neurodevelopmental disorders may persist throughout adulthood and place economic burdens on society (Landrigan et al. 2012). Previous studies indicate that environmental factors may play a role in the etiology of these disorders (Bouchard et al. 2010; Braun et al. 2008; Ciesielski et al. 2012; Froehlich et al. 2009; London et al. 2012). In our study, concentrations of 3-PBA were not associated with significantly higher odds of parent-reported LD and/or ADHD in children 6–15 years of age from a representative sample of the U.S. general population after controlling for covariates; this finding remained after controlling for creatinine and other environmental chemicals previously linked to altered neurodevelopment. Also, detection of *cis-* and *trans*-DCCA was not associated with any of our outcomes.

To our knowledge, only two studies have assessed the association between postnatal pyrethroid exposure and children’s neurodevelopment. Similar to a recent Canadian population-based study in 6- to 11-year-old children (Oulhote and Bouchard 2013), no significant associations were observed between 3-PBA concentrations and parent-reported behavior problems in our population. However, contrary to the Canadian study, which reported an association between urinary concentrations of *cis*-DCCA and parent-reported behavioral problems (as assessed using a validated questionnaire encompassing various dimension scales such as emotional symptoms, conduct problems, hyperactivity/inattention, and peer problems) (Oulhote and Bouchard 2013), we did not observe significant associations between detection of *cis-*DCCA and parent-reported LD and/or ADHD in our study population. There were key differences in these two studies that may limit comparisons: Detection frequencies in the Canadian study were higher (35.6% in NHANES vs. 97.3% in the Canadian children) due to lower limits of detection (LOD = 0.10 μg/L in NHANES vs. 0.007 μg/L in Canadian study); the years during which samples were collected were later in the Canadian study (1999–2002 for NHANES vs. 2007–2009 in the Canadian study); and the outcomes assessed differed. In the Nicaraguan study (Rodríguez 2012), which reported an association between 3-PBA concentrations and ADHD in girls, the children lived in an agricultural community where pyrethroid exposures were more than seven times higher than those reported in our population (GM = 2.8 μg/g creatinine; *n* = 74 vs. GM = 0.37 μg/ creatinine; *n* = 1,861, respectively); sampling of urine samples occurred at a later time (2008 vs. 1999–2002 in NHANES); and the mixture of pyrethroids used may have differed (Rodríguez 2012).

Despite the scarcity of epidemiologic data on associations between pyrethroid insecticides and children’s neurobehavioral development, animal studies have suggested potential mechanisms of action. Behavioral changes and neurochemical changes to cholinergic, dopaminergic, and catecholaminergic pathways have been reported at subacute and subchronic doses in rodent studies for select pyrethroid insecticides (Aziz et al. 2001; Elwan et al. 2006; Eriksson and Fredriksson 1991; Lazarini et al. 2001; Shafer et al. 2005). A systematic review of developmental *in vivo* toxicity studies of pyrethroids by Shafer et al. (2005) highlights changes in motor activity and in muscarinic acetylcholine receptor density in rodents. One study by Sinha et al. (2006) reported that pyrethroid exposure in rat pups during the prenatal, perinatal, and early postnatal period at doses meant to simulate those experienced during human residential use led to significant oxidative stress, an increase in lipid peroxidation, and a decrease in antioxidants, glutathione, superoxide dismutase, and catalase in various brain areas. The hippocampus was the most affected region. A decrease in learning and memory performance was reported to accompany these neurochemical changes, suggesting that pyrethroid exposure during early life adversely affects the developing brain by causing cholinergic dysfunction and leading to deficits in learning and memory (Sinha et al. 2006). Significant and persistent memory and learning deficiencies were also linked to muscarinic receptor binding in young rats after low-level prenatal pyrethroid exposure (Aziz et al. 2001). Additionally, significant dopaminergic changes have been reported from early-life exposure to multiple pyrethroids in rodents (Carloni et al. 2013; Elwan et al. 2006), and recent experimental studies suggest that dopamine may play a role in ADHD through abnormal functioning of dopaminergic receptors (Wu et al. 2012). High-dose pubertal pyrethroid exposure has also been linked to hormone disruption (testosterone and estradiol synthesis) and expression of androgen receptor in the cerebral cortex in mice; such changes in the developing brain may be deleterious for neurobehavioral development (Liu et al. 2011).

The present study has several limitations. The main limitation is that NHANES is a cross-sectional survey, so it was not possible to determine the temporal sequence of exposure and outcome and to establish causality. Reverse causality is conceivable—that is, children with behavioral problems such as ADHD by virtue of these problems may become more exposed; for example, their higher activity levels may lead to more exposure. It also remains possible that uncontrolled confounding may be biasing our results toward the null (e.g., if a better-educated parent were more likely to have their child diagnosed and less likely to use pyrethroids at home). Although we tried to adjust for several important confounders, including education and health insurance status, we were limited by the variables available to us in this national survey.

There are also a number of limitations with the biomarkers of exposure used. Pyrethroids are nonpersistent and thus rapidly metabolized in the body, so the use of single spot urine samples may not accurately reflect typical exposure levels. Additionally, the use of nonspecific urinary metabolites to assess pyrethroid exposure and the fact that metabolite concentrations in urine may also reflect direct exposure to the preformed breakdown products (i.e., the metabolites themselves) present in the environment (CDC 2009; Starr et al. 2008) prevents us from determining associations with specific pyrethroid insecticide(s). Also, the high detection limits and low detection frequencies of *cis-* and *trans-*DCCA did not allow us to assess the association between actual metabolite concentrations and our outcomes. Low percent of detectable concentrations for these metabolites limited our sample size in that stratum, and thus our ability to find an association. Additionally, exposure to pyrethroids during a potentially critical period of vulnerability—prenatally and during infancy—was not assessed in NHANES.

Urine collection in our study population took place before the residential phase-out of other pesticides that are being replaced by pyrethroid insecticides; thus, residential use of pyrethroids may not have been widespread. In fact, the GM concentration for 3-PBA for U.S. children 6–15 years of age with data from the NHANES 2007–2008 (CDC 2013) was higher than that observed during 1999–2002, the period of the present study (GM = 0.42 μg/L vs. GM = 0.32 μg/L, respectively). Unfortunately, data on our outcomes of interest were not available for 2007–2008. Higher 3-PBA levels were also noted in two later children’s studies: One 2003–2004 study conducted in Seattle, Washington, of children 3–11 years of age reported a median 3-PBA concentration nearly four times higher than that of our study population (1.2 μg/L vs. 0.31 μg/L) (Morgan 2012); and another study in 2009 of farmworker children 2–8 years of age found 3-PBA concentrations five times higher than those in our study population (GM = 1.97 μg/g creatinine vs. GM = 0.37 μg/g creatinine, respectively) (Trunnelle et al. 2014).

Another study limitation is that “diagnosis” of LD and ADHD was based on parental/guardian report of clinical or teacher evaluation rather than based on school or medical records or a neuropsychological assessment. However, to increase the likelihood that a child was indeed an ADHD case, we also included children who were taking stimulant medication, and the results remained nonsignificant (i.e., ORs were further attenuated). Furthermore, we examined only the association between pyrethroid exposure and LD and ADHD, but there may be other childhood behavioral disorders (e.g., anxiety, aggression) that are related to pyrethroid exposure that we were not able to investigate with the available data.

Despite these limitations, our analyses have certain strengths. First, we conducted our analyses on a large, representative sample of children from the U.S. general population. Thus, results are generalizable to U.S. children 6–15 years of age during the time period assessed. To our knowledge, this is also the first study in the United States to assess the association between postnatal pyrethroid exposure and altered neurodevelopment in children. We also took into account numerous potential confounders as well as other environmental exposures previously linked to the outcomes of interest.

In conclusion, postnatal pyrethroid exposure was not significantly associated with parent-reported LD, ADHD, or both LD and ADHD in children 6–15 years of age participating in NHANES during 1999–2002. However, the cross-sectional nature of this study does not allow us to establish causality nor to examine exposure during the potentially more critical prenatal period. We found that a large percentage of children participating in NHANES in 1999–2002 had detectable levels of the pyrethroid metabolite 3-PBA; however, recent studies indicate that pyrethroid use has increased since the residential phase-out of certain organophosphate insecticides (Gan 2008; Horton et al. 2011a; Power and Sudakin 2007), and higher urinary pyrethroid concentrations have been reported in samples collected after 2002 (Morgan 2012; Trunnelle et al. 2014). Given that pyrethroid use is increasing and is widespread, future research should evaluate the safety of current levels of pyrethroid insecticide exposures during critical windows of brain development.

## Supplemental Material

(482 KB) PDF

## References

[r1] American Psychiatric Association. (2013). Diagnostic and Statistical Manual of Mental Disorders, 5th Edition (DSM–5).

[r2] ATDSR (Agency for Toxic Substances and Disease Registry). (2003). Toxicological Profile for Pyrethrins and Pyrethroids.. http://www.atsdr.cdc.gov/toxprofiles/tp155.pdf.

[r3] Aziz MH, Agrawal AK, Adhami VM, Shukla Y, Seth PK (2001). Neurodevelopmental consequences of gestational exposure (GD14–GD20) to low dose deltamethrin in rats.. Neurosci Lett.

[r4] Baker SE, Olsson AO, Barr DB (2004). Isotope dilution high-performance liquid chromatography–tandem mass spectrometry method for quantifying urinary metabolites of synthetic pyrethroid insecticides.. Arch Environ Contam Toxicol.

[r5] Barr DB Olsson AO Wong LY Udunka S Baker SE Whitehead RD 2010 Urinary concentrations of metabolites of pyrethroid insecticides in the general U.S. population: National Health and Nutrition Examination Survey 1999–2002. Environ Health Perspect 118 742 748; 10.1289/ehp.0901275 20129874 PMC2898848

[r6] Barr D Wilder L Caudill S Gonzalez A Needham L Pirkle J 2005 Urinary creatinine concentrations in the U.S. population: implications for urinary biologic monitoring measurements. Environ Health Perspect 113 192 200; 10.1289/ehp.7337 15687057 PMC1277864

[r7] Bouchard MF, Bellinger DC, Wright RO, Weisskopf MG (2010). Attention–deficit/hyperactivity disorder and urinary metabolites of organophosphate pesticides.. Pediatrics.

[r8] Bouchard MF Chevrier J Harley KG Kogut K Vedar M Calderon N 2011 Prenatal exposure to organophosphate pesticides and IQ in 7–year–old children. Environ Health Perspect 119 1189 1195; 10.1289/ehp.1003185 21507776 PMC3237357

[r9] Braun JM Froehlich TE Daniels JL Dietrich KN Hornung R Auinger P 2008 Association of environmental toxicants and conduct disorder in U.S. children: NHANES 2001–2004. Environ Health Perspect 116 956 962; 10.1289/ehp.11177 18629321 PMC2453167

[r10] Cantalamessa F (1993). Acute toxicity of two pyrethroids, permethrin, and cypermethrin in neonatal and adult rats.. Arch Toxicol.

[r11] Carloni M, Nasuti C, Fedeli D, Montani M, Vadhana MS, Amici A (2013). Early life permethrin exposure induces long-term brain changes in Nurr1, NF-kB and Nrf-2.. Brain Res.

[r12] CDC (Centers for Disease Control and Prevention). (2009). Fourth Report on Human Exposure to Environmental Chemicals.. http://www.cdc.gov/exposurereport/pdf/FourthReport.pdf.

[r13] CDC (Centers for Disease Control and Prevention). (2013). NHANES 2007–2008 Laboratory Data. Urinary Pyrethroids, Herbicides, and OP Metabolites (Subsample).. http://wwwn.cdc.gov/nchs/nhanes/search/datapage.aspx?Component=Laboratory&CycleBeginYear=2007.

[r14] CDC (Centers for Disease Control and Prevention). (2014). About the National Health and Nutrition Examination Survey.. http://www.cdc.gov/nchs/nhanes/about_nhanes.htm.

[r15] Ciesielski T Weuve J Bellinger DC Schwartz J Lanphear B Wright RO 2012 Cadmium exposure and neurodevelopmental outcomes in U.S. children. Environ Health Perspect 120 758 763; 10.1289/ehp.1104152 22289429 PMC3346779

[r16] Elwan MA, Richardson JR, Guillot TS, Caudle WM, Miller GW (2006). Pyrethroid pesticide-induced alterations in dopamine transporter function.. Toxicol Appl Pharm.

[r17] Eriksson P, Fredriksson A (1991). Neurotoxic effects of two different pyrethroids, bioallethrin and deltamethrin, on immature and adult mice: changes in behavioral and muscarinic receptor variables.. Toxicol Appl Pharm.

[r18] Farag AT, Goda NF, Shaaban NA, Mansee AH (2007). Effects of oral exposure of synthetic pyrethroid, cypermethrin on the behavior of F1-progeny in mice.. Reprod Toxicol.

[r19] Froehlich TE, Lanphear BP, Auinger P, Hornung R, Epstein JN, Braun J (2009). Association of tobacco and lead exposures with attention-deficit/hyperactivity disorder.. Pediatrics.

[r20] Gan J. (2008). Synthetic Pyrethroids: Occurrence and Behavior in Aquatic Environments.

[r21] Horton MK, Jacobson JB, McKelvey W, Holmes D, Fincher B, Quantano A (2011a). Characterization of residential pest control products used in inner city communities in New York City.. J Expo Sci Environ Epidemiol.

[r22] Horton MK, Rundle A, Camann DE, Boyd Barr D, Rauh VA, Whyatt RM (2011b). Impact of prenatal exposure to piperonyl butoxide and permethrin on 36-month neurodevelopment.. Pediatrics.

[r23] Landrigan PJ Lambertini L Birnbaum LS 2012 A research strategy to discover the environmental causes of autism and neurodevelopmental disabilities [Editorial]. Environ Health Perspect 120 A258 A260; 10.1289/ehp.1104285 22543002 PMC3404655

[r24] Lanphear BP, Dietrich K, Auinger P, Cox C (2000). Cognitive deficits associated with blood lead concentrations < 10 microg/dL in US children and adolescents.. Public Health Rep.

[r25] Lazarini CA, Florio JC, Lemonica IP, Bernardi MM (2001). Effects of prenatal exposure to deltamethrin on forced swimming behavior, motor activity, and striatal dopamine levels in male and female rats.. Neurotox Teratol.

[r26] Liu P, Meng XH, Wang H, Ji YL, Zhao M, Zhao XF (2011). Effects of pubertal fenvalerate exposure on testosterone and estradiol synthesis and the expression of androgen and estrogen receptors in the developing brain.. Toxicol Lett.

[r27] London L, Beseler C, Bouchard MF, Bellinger DC, Colosio C, Grandjean P (2012). Neurobehavioral and neurodevelopmental effects of pesticide exposures.. Neurotoxicology.

[r28] Lu C, Barr DB, Pearson MA, Walker LA, Bravo R (2009). The attribution of urban and suburban children’s exposure to synthetic pyrethroid insecticides: a longitudinal assessment.. J Expo Sci Environ Epidemiol.

[r29] Lubin JH Colt JS Camann D Davis S Cerhan JR Severson RK 2004 Epidemiologic evaluation of measurement data in the presence of detection limits. Environ Health Perspect 112 1691 1696; 10.1289/ehp.7199 15579415 PMC1253661

[r30] Mandhane SN, Chopde CT (1997). Neurobehavioral effects of low level fenvalerate exposure in mice.. Indian J Exp Biol.

[r31] Meng XH, Liu P, Wang H, Zhao XF, Xu ZM, Chen GH (2011). Gender-specific impairments on cognitive and behavioral development in mice exposed to fenvalerate during puberty.. Toxicol Lett.

[r32] Morgan MK (2012). Children’s exposures to pyrethroid insecticides at home: a review of data collected in published exposure measurement studies conducted in the United States.. Int J Environ Res Public Health.

[r33] Nasuti C, Cantalamessa F, Falcioni G, Gabbianelli R (2003). Different effects of Type I and Type II pyrethroids on erythrocyte plasma membrane properties and enzymatic activity in rats.. Toxicology.

[r34] Olsson AO, Baker SE, Nguyen JV, Romanoff LC, Udunka SO, Walker RD (2004). A liquid chromatography–tandem mass spectrometry multiresidue method for quantification of specific metabolites of organophosphorus pesticides, synthetic pyrethroids, selected herbicides, and DEET in human urine.. Anal Chem.

[r35] Oulhote Y Bouchard M 2013 Urinary metabolites of organophosphate and pyrethroid pesticides and behavioral problems in Canadian children. Environ Health Perspect 121 1378 1384; 10.1289/ehp.1306667 24149046 PMC3855516

[r36] Pastor PN, Reuben CA. (2002). Attention Deficit Disorder and Learning Disability: United States, 1997–98. Vital Health Stat 10(206).. http://www.eric.ed.gov/PDFS/ED467266.pdf.

[r37] Power LE, Sudakin DL (2007). Pyrethrin and pyrethroid exposures in the United States: a longitudinal analysis of incidents reported to poison centers.. J Med Toxicol.

[r38] Ray DE, Forshaw PJ (2000). Pyrethroid insecticides: poisoning syndromes, synergies, and therapy.. J Toxicol Clin Toxicol.

[r39] Rodríguez T. (2012). Environmental Pesticide Exposure and Neurobehavioral Effects among Children of Nicaraguan Agricultural Workers [PhD Dissertation]. Uppsala, Sweden:Uppsala University.. http://www.diva-portal.org/smash/get/diva2:558620/FULLTEXT01.pdf.

[r40] Shafer TJ Meyer DA Crofton KM 2005 Developmental neurotoxicity of pyrethroid insecticides: critical review and future research needs. Environ Health Perspect 113 123 136; 10.1289/ehp.7254 15687048 PMC1277854

[r41] Sinha C, Seth K, Islam F, Chaturvedi RK, Shukla S, Mathur N (2006). Behavioral and neurochemical effects induced by pyrethroid-based mosquito repellent exposure in rat offsprings during prenatal and early postnatal period.. Neurotoxicol Teratol.

[r42] Soderlund DM, Clark JM, Sheets LP, Mullin LS, Piccirillo VJ, Sargent D (2002). Mechanisms of pyrethroid neurotoxicity: implications for cumulative risk assessment.. Toxicology.

[r43] Starr J, Graham S, Stout D, Andrews K, Nishioka M (2008). Pyrethroid pesticides and their metabolites in vacuum cleaner dust collected from homes and day-care centers.. Environ Res.

[r44] Trunnelle KJ, Bennett DH, Ahn KC, Schenker MB, Tancredi DJ, Gee SJ (2014). Concentrations of the urinary pyrethroid metabolite 3-phenoxybenzoic acid in farm worker families in the MICASA study.. Environ Res.

[r45] U.S. EPA (U.S. Environmental Protection Agency). (2013a). Permethrin, Resmethrin, d-Phenothrin (Sumithrin®): Synthetic Pyrethroids for Mosquito Control.. http://www2.epa.gov/mosquitocontrol/permethrin-resmethrin-d-phenothrin-sumithrinr-synthetic-pyrethroids-mosquito-control.

[r46] U.S. EPA (U.S. Environmental Protection Agency). (2013b). Pyrethroids and Pyrethrins.. http://www.epa.gov/oppsrrd1/reevaluation/pyrethroids-pyrethrins.html.

[r47] Wei B, Mohan KR, Weisel CP (2012). Exposure of flight attendants to pyrethroid insecticides on commercial flights: urinary metabolite levels and implications.. Int J Hyg Environ Health.

[r48] Williams MK Rundle A Holmes D Reyes M Hoepner LA Barr DB 2008 Changes in pest infestation levels, self-reported pesticide use, and permethrin exposure during pregnancy after the 2000–2001 U.S. Environmental Protection Agency restriction of organophosphates. Environ Health Perspect 116 1681 1688; 10.1289/ehp.11367 19079720 PMC2599763

[r49] Wu J, Xiao H, Sun H, Zou L, Zhu LQ (2012). Role of dopamine receptors in ADHD: a systematic meta-analysis.. Mol Neurobiol.

[r50] Xue Z, Li X, Su Q, Xu L, Zhang P, Kong Z (2013). Effect of synthetic pyrethroid pesticide exposure during pregnancy on the growth and development of infants.. Asia Pac J Public Health.

[r51] Zipf G, Chiappa M, Porter KS, Ostchega Y, Lewis BG, Dostal J. (2013). National Health and Nutrition Examination Survey: Plan and Operations, 1999–2010. Vital Health Stat 1(56).. http://www.cdc.gov/nchs/data/series/sr_01/sr01_056.pdf.

