# Supplementary material for: Pyrethroid Pesticide Exposure and Parental Report of Learning Disability and Attention Deficit/Hyperactivity Disorder in U.S. Children: NHANES 1999–2002
Source: Environ Health Perspect. 2014 Sep 5;122(12):1336–42. doi: 10.1289/ehp.1308031 (PMC4256700; doi:10.1289/ehp.1308031)
Supplement: (482 KB) PDF [file ehp.1308031.s001.508.pdf]

Environmental Health Perspectives doi:10.1289/ehp.1308031

Supplemental Material

Pyrethroid Pesticide Exposure and Parental Report of Learning Disability and Attention Deficit/Hyperactivity Disorder in U.S. Children: NHANES 1999–2002

Lesliam Quirós-Alcalá, Suril Mehta, and Brenda Eskenazi

Table S1. Demographic/personal characteristics and environmental factors for study participants with and without pyrethroid metabolite data [n (%)].[a]

Children's characteristics	All children n = 4672	Children with 3-PBA (n = 1861)	Children without 3-PBA (n = 2811)	Children with *cis*-DCCA (n = 1839)	Children without *cis*-DCCA (n = 2833)	Children with *trans*-DCCA (n = 1848)	Children without *trans*-DCCA (n = 2824)
**Gender**							
Male	2308 (51.4)	899 (51.7)	1409 (52.1)	885 (51.4)	1423 (52.2)	891 (51.6)	1417 (52.1)
Female	2364 (48.6)	962 (48.3)	1402 (47.9)	954 (48.6)	1410 (47.8)	957 (48.4)	1407 (47.9)
Missing	0	0	0	0	0	0	0
**Race/ethnicity**							
Non-Hispanic White	1201 (60.0)	466 (58.5)	735 (61.7)	460 (58.5)	741 (61.7)	462 (58.4)	739 (61.7)
Non-Hispanic Black	1450 (14.9)	596 (14.6)	854 (14.3)	594 (14.7)	856 (14.2)	590 (14.5)	860 (14.3)
Mexican American	1639 (11.6)	625 (11.3)	1014 (11.8)	612(11.1)	1027(11.9)	623 (11.4)	1016 (11.7)
Other	383 (13.5)	174 (15.7)	208 (12.2)	173 (15.7)	209 (12.2)	173 (15.7)	209 (12.2)
Missing	0	0	0	0	0	0	0
**Poverty-income-ratio (PIR)**							
< 0.94	1323 (21.7)	549 (22.9)	774 (20.6)	540 (22.7)	783 (20.7)	542 (22.7)	781 (20.7)
0.94-1.80	1096 (23.5)	441 (24.0)	655 (23.0)	435 (23.9)	661 (23.0)	439 (24.0)	657 (23.0)
1.81-3.56	992 (27.1)	387 (25.9)	605 (28.1)	382 (25.9)	610 (28.1)	384 (25.9)	608 (28.1)
> 3.56	810 (27.7)	318 (27.2)	492 (28.4)	317 (27.5)	493 (28.2)	318 (27.4)	492 (28.2)
Missing	451	166	285	165	286	165	286
**Household reference person's education level**							
< High school	1750 (24.2)	688 (24.7)	1062 (23.9)	678 (24.7)	1072 (23.8)	683 (24.7)	1067 (23.8)
High school	1113 (27.7)	438 (25.9)	675 (28.8)	431 (25.6)	682 (29.1)	434 (25.7)	679 (29.0)
> High school	1637 (48.1)	663 (49.4)	974 (47.3)	658 (49.7)	979 (47.1)	659 (49.6)	978 (47.2)
Missing	172	72	100	72	100	72	100
**Low birth weight (< 2500g)**							
Yes	397 (7.5)	160 (7.4)	239 (7.7)	155 (7.3)	242 (7.7)	157 (7.3)	240 (7.6)
No	4077 (92.5)	1629 (92.6)	2446 (92.3)	1613 (92.7)	2464 (92.3)	1621 (92.7)	2456 (92.4)
Missing	198	72	126 (4.5)	71	127	70	128
**Age**							
6-7 years	751 (21.0)	333 (19.8)	418 (20.2)	326 (19.5)	425 (20.4)	329 (19.6)	422 (20.3)
8-9 years	771 (19.7)	377 (20.4)	394 (17.3)	374 (20.5)	397 (17.3)	277 (20.5)	394 (17.2)
10-11 years	737 (19.7)	339 (18.3)	398 (18.9)	334 (18.3)	403 (18.9)	334 (18.2)	403 (19.0)
12-13 years	1251 (20.0)	411 (20.6)	840 (22.2)	406 (20.6)	845 (22.1)	408 (20.1)	843 (22.1)
14-15 years	1162 (19.6)	401 (20.9)	761 (21.4)	399 (21.1)	763 (21.3)	400 (21.1)	762 (21.3)
Missing	0	0	0	0	0	0	0
**Neonatal intensive care unit (NICU) admission**							
Yes	516 (11.7)	205 (12.0)	311 (11.3)	204 (12.1)	312 (11.3)	203 (12.0)	313 (11.3)
No	4115 (88.3)	1641 (88.0)	2474 (88.7)	1620 (87.9)	2495 (88.7)	1630 (88.0)	2485 (88.7)
Missing	41	15	26	15	26	15	26
**Attended daycare or preschool**							
Yes	3077 (72.9)	1229 (71.6)	1848 (73.9)	1219 (71.9)	1858 (73.7)	1224 (72.0)	1853 (73.6)
No	1588 (27.1)	630 (28.4)	958 (26.1)	618 (28.1)	970 (26.3)	622 (28.0)	966 (26.4)
Missing	7	2	5	2	5	2	5

Children's characteristics	All children n = 4672	Children with 3-PBA (n = 1861)	Children without 3-PBA (n = 2811)	Children with *cis*-DCCA (n = 1839)	Children without *cis*-DCCA (n = 2833)	Children with *trans*-DCCA (n = 1848)	Children without *trans*-DCCA (n = 2824)
**Health insurance**							
Yes	3774 (81.2)	1492 (82.8)	2282 (88.7)	1474 (82.7)	2300 (88.7)	1479 (82.7)	2295 (88.7)
No	837 (13.8)	348 (17.2)	489 (11.3)	344 (17.3)	493 (11.3)	348 (17.3)	489 (11.3)
Missing	61	21	40	21	40	21	40
**Total DAPs**							
< 3.15×10^{-8} mol/L	517 (30.3)	517 (30.5)	0	512(30.3)	5 (60.1)	515 (30.4)	2 (46.5)
3.15×10^{-8} to 1.14×10^{-7}mol/L	594 (31.0)	594 (31.1)	0	589 (31.2)	5 (15.3)	592 (31.2)	2 (6.7)
> 1.14×10^{-7} mol/L	719 (38.7)	718 (38.4)	1 (100)	712 (38.5)	7 (25.6)	715 (38.4)	4 (46.8)
Missing	2842	32	2810	26	2816	26	2816
**Blood lead level**							
0.2-1.2 µg/dL	2008 (52.8)	771 (50.1)	1237 (55.6)	762 (50.2)	1246 (55.5)	769 (50.3)	1239 (55.4)
1.3-2.1 µg/dL	1265 (30.5)	522 (31.4)	743 (29.5)	516 (31.5)	749 (29.4)	519 (31.5)	746 (29.4)
> 2.1 µg/dL	862 (16.7)	369 (18.5)	493 (14.9)	363 (18.3)	499 (15.1)	363 (18.2)	499 (15.2)
Missing	537	199	338	198	339	197	340
**Serum cotinine**							
Lower 50 (< 0.06 ng/ml)	1670 (42.9)	650 (41.3)	1020 (44.2)	638 (41.4)	1032 (44.0)	649 (41.6)	1021 (43.9)
Upper 50 (0.06-9.99 ng/mL) ng/ml)	2210 (54.5)	910 (55.7)	1300 (53.2)	902 (55.5)	1308 (53.4)	901 (55.3)	1309 (53.5)
≥ 10 ng/ml	91 (2.6)	35 (3.1)	56 (2.6)	35 (3.1)	56 (2.5)	35 (3.1)	56 (2.6)
Missing	701	266	435	264	437	263	438
**Creatinine**							
< 92 mg/dL	1481 (37.2)	685 (38.8)	796 (34.7)	673 (38.6)	808 (34.9)	680 (38.6)	801 (34.9)
92-164 mg/dL	1742 (39.3)	717 (39.3)	1025 (39.1)	709 (39.3)	1033 (39.1)	713 (39.4)	1029 (39.0)
> 164 mg/dL	1276 (23.5)	459 (21.8)	817 (26.2)	457 (22.0)	819 (26.1)	455 (21.9)	821 (26.1)
Missing	173	0	173	0	173	0	173
**Mother's age at birth**							
< 25 years	2215 (41.7)	861 (41.8)	1354 (41.4)	853 (41.9)	1362 (41.3)	856 (42.0)	1359 (41.3)
25–34 years	2015 (47.8)	836 (49.2)	1179 (47.1)	822 (49.0)	1193 (47.3)	829 (48.9)	1186 (47.3)
35+ years	373 (10.5)	135 (9.0)	238 (11.5)	135 (9.1)	238 (11.4)	135 (9.1)	238 (11.4)
Missing	69	29	40	29	40	28	41
**Mother smoked during pregnancy**							
Yes	647 (18.3)	265 (18.7)	382 (18.1)	263 (18.7)	384 (18.1)	261 (18.7)	386 (18.1)
No	3952 (81.7)	1566 (81.3)	2386 (81.9)	1546 (81.3)	2406 (81.9)	1557 (81.3)	2395 (81.9)
Missing	73	30	43	30	43	30	43

[a]Percent values provided in parentheses are weighted.

Table S2. Prevalence of outcomes for study participants with and without pyrethroid urinary metabolite data [n (%)].

Neurodevelopmental outcome	All children (n = 4672)	Children with 3-PBA (n = 1861)	Children without 3-PBA (n = 2811)	Children with *cis*-DCCA (n = 1839)	Children without *cis*-DCCA (n = 2833)	Children with *trans*-DCCA (n = 1848)	Children without *trans*-DCCA (n = 2824)
**LD**							
Yes	544 (11.5)	223 (12.7)	321 (11.2)	221 (12.8)	323 (11.1)	221 (12.7)	323 (11.2)
No	4118 (88.5)	1636 (87.3)	2482 (88.8)	1616 (87.2)	2502 (88.9)	1625 (87.3)	2493 (88.8)
Missing	10	2	8	2	8	2	8
**ADHD**							
Yes	342 (9.3)	148 (10.0)	194 (9.1)	146 (9.9)	196 (9.2)	147 (9.9)	195 (9.2)
No	4320 (90.7)	1708 (90.0)	2612 (90.9)	1688 (90.1)	2632 (90.8)	1697 (90.1)	2623 (90.8)
Missing	10	5	5	5	5	4	6
**LD+ADHD**							
Yes	174 (4.3)	78 (5.4)	96 (3.8)	77 (5.4)	97 (3.8)	78 (5.4)	96 (3.8)
No	4479 (95.7)	1776 (94.6)	2703 (96.2)	1755 (94.6)	2724 (96.2)	1764 (94.6)	2715 (96.2)
Missing	19	7	12	7	12	6	13

Table S3. ORs for LD, ADHD, and both LD and ADHD for every 10-fold increase in urinary 3-PBA concentrations including and excluding select variables as a covariates in the final models.[a,b]

Neurodevelopmental outcome	cOR (95% CI)	*p*-value	aOR (95% CI)	*p*-value	aOR_{creat} (95% CI)	*p*-value
**Including PIR (n = 1551)**						
LD	1.18 (0.90, 1.56)	0.24	1.08 (0.80, 1.48)	0.61	1.00 (0.70, 1.41)	0.98
ADHD	1.18 (0.84, 1.66)	0.34	1.07 (0.74, 1.57)	0.72	0.98 (0.65, 1.46)	0.90
LD+ADHD	1.43 (0.86, 2.36)	0.17	1.24 (0.74, 2.07)	0.42	1.10 (0.64, 1.87)	0.73
**Excluding PIR (n = 1551)**						
LD	1.18 (0.90, 1.56)	0.23	1.13 (0.83, 1.54)	0.43	1.04 (0.73, 1.48)	0.82
ADHD	1.18 (0.84, 1.66)	0.34	1.09 (0.76, 1.56)	0.65	0.99 (0.66, 1.46)	0.94
LD+ADHD	1.43 (0.86, 2.36)	0.17	1.32 (0.76, 2.28)	0.32	1.16 (0.65, 2.05)	0.62
**Including total DAPs (n = 1652)**						
LD	1.27 (1.02, 1.58)	0.04	1.16 (0.90, 1.50)	0.25	1.11 (0.82, 1.49)	0.50
ADHD	1.26 (0.96, 1.64)	0.09	1.11 (0.81, 1.51)	0.52	1.05 (0.75, 1.46)	0.79
LD+ADHD	1.55 (1.04, 2.32)	0.03	1.33 (0.85, 2.08)	0.21	1.26 (0.78, 2.03)	0.34
**Excluding total DAPs (n = 1652)**						
LD	1.27 (1.02, 1.58)	0.04	1.19 (0.93, 1.53)	0.17	1.11 (0.83, 1.49)	0.47
ADHD	1.26 (0.96, 1.64)	0.09	1.15 (0.85, 1.57)	0.36	1.06 (0.76, 1.47)	0.73
LD+ADHD	1.55 (1.04, 2.32)	0.03	1.45 (0.92, 2.26)	0.11	1.30 (0.82, 2.08)	0.26
**Including blood lead (n = 1511)**						
LD	1.32 (1.07, 1.64)	0.01	1.23 (0.95, 1.59)	0.11	1.16 (0.87, 1.57)	0.31
ADHD	1.36 (1.05, 1.78)	0.02	1.24 (0.93, 1.64)	0.14	1.18 (0.87, 1.59)	0.30
LD+ADHD	1.78 (1.22, 2.58)	0.003	1.63 (1.01, 2.65)	0.05	1.49 (0.89, 2.51)	0.13
**Excluding blood lead (n = 1511)**						
LD	1.32 (1.07, 1.64)	0.01	1.26 (0.97, 1.65)	0.09	1.20 (0.88, 1.64)	0.25
ADHD	1.36 (1.05, 1.78)	0.02	1.26 (0.94, 1.68)	0.12	1.20 (0.88, 1.64)	0.25
LD+ADHD	1.78 (1.22, 2.58)	0.003	1.74 (1.09, 2.78)	0.02	1.61 (0.99, 2.62)	0.06
**Including serum cotinine (n = 1451)**						
LD	1.32 (1.07, 1.62)	0.01	1.24 (0.95, 1.61)	0.12	1.17 (0.87, 1.59)	0.30
ADHD	1.38 (1.05, 1.83)	0.02	1.27 (0.92, 1.76)	0.15	1.21 (0.85, 1.72)	0.28
LD+ADHD	1.77 (1.22, 2.57)	0.003	1.73 (1.08, 2.77)	0.02	1.60 (0.97, 2.63)	0.06
**Excluding serum cotinine (n = 1451)**						
LD	1.32 (1.07, 1.62)	0.01	1.28 (0.98, 1.66)	0.07	1.21 (0.86, 1.65)	0.23
ADHD	1.38 (1.05, 1.83)	0.02	1.30 (0.95, 1.79)	0.10	1.24 (0.88, 1.75)	0.22
LD+ADHD	1.77 (1.22, 2.57)	0.003	1.78 (1.13, 2.81)	0.01	1.63 (1.00, 2.66)	0.05
**Including all chemicals and PIR (n = 1333)**						
LD	1.28 (1.01, 1.61)	0.03	1.14 (0.85, 1.52)	0.39	1.05 (0.76, 1.46)	0.76
ADHD	1.32 (0.96, 1.81)	0.09	1.16 (0.81, 1.65)	0.42	1.11 (0.76, 1.60)	0.59
LD+ADHD	1.66 (1.11, 2.47)	0.01	1.33 (0.84, 2.13)	0.23	1.21 (0.76, 1.92)	0.43
**Excluding all chemicals and PIR (n = 1333)**						
LD	1.28 (1.01, 1.61)	0.03	1.25 (0.94, 1.66)	0.12	1.16 (0.83, 1.61)	0.40
ADHD	1.32 (0.96, 1.81)	0.09	1.23 (0.88, 1.73)	0.23	1.16 (0.80, 1.68)	0.44
LD+ADHD	1.66 (1.11, 2.47)	0.01	1.65 (1.04, 2.61)	0.03	1.45 (0.91, 2.33)	0.12

Abbreviations: cOR=Crude odds ratio; aOR=Adjusted odds ratio; aOR_{creat}=Adjusted odds ratio including urine creatinine concentration as a covariate in the model.

[a]Sample size for each model was based on the number of children with complete information on exposure, outcome, and covariates. [b]Models were adjusted for sex, age, race/ethnicity, household reference education level, low birth weight status, maternal age at child's birth, NICU admission, maternal smoking during pregnancy, daycare/preschool attendance, and health insurance.

Table S4. Pyrethroid metabolite descriptive statistics by NHANES cycle year for children 6 to 15 years of age.

Pyrethroid metabolite	Cycle year 1999-2000	Cycle year 2000-2001
**3-PBA**		
N	841	1020
N (%) with detectable concentrations	623 (74.1)	811 (79.5)
GM (95% CI) (µg/L)	0.35 (0.24, 0.50)	0.29 (0.23, 0.38)
***p*-value comparing GM**[a]	0.42	
***cis*-DCCA**		
N	819	1020
N (%) with detectable concentrations	349 (42.6)	305 (29.9)
***p*-value**[b]	0.0001	
***trans*-DCCA**		
N	833	1015
N (%) with detectable concentrations	322 (38.7)	304 (30.0)
***p*-value**[b]	0.006	

[a]T-test p-value comparing 3-PBA concentrations between cycle year. [b]Chi-square p-value comparing detection rates of *cis*- and *trans*-DCCA by cycle year.
